# The natural history of Canavan disease: 23 new cases and comparison with patients from literature

**DOI:** 10.1186/s13023-020-01659-3

**Published:** 2021-05-19

**Authors:** Annette Bley, Jonas Denecke, Alfried Kohlschütter, Gerhard Schön, Sandra Hischke, Philipp Guder, Tatjana Bierhals, Heather Lau, Maja Hempel, Florian S. Eichler

**Affiliations:** 1 Department of Pediatrics, University Medical Center Hamburg Eppendorf, Martini-Str. 52, 20246 Hamburg, Germany; 2 Department of Neurogenetics, NYU, 222 East 41st Street, New York, NY 10017 USA; 3 Institute of Medical Biometry and Epidemiology, University Medical Center Hamburg Eppendorf, Martini-Str. 52, 20246 Hamburg, Germany; 4 Department of Human Genetics, University Medical Center Hamburg Eppendorf, Martini-Str. 52, 20246 Hamburg, Germany; 5 Department of Neurology, MGH, Harvard Medical School, 55 Fruit St, Boston, MA 02114 USA

**Keywords:** Leukodystrophy, Early childhood neurodegeneration, Aspartoacylase deficiency, Spongy degeneration of the brain, Canavan-van bogaert-bertrand disease

## Abstract

**Background:**

Canavan disease (CD, MIM # 271900) is a rare and devastating leukodystrophy of early childhood. To identify clinical features that could serve as endpoints for treatment trials, the clinical course of CD was studied retrospectively and prospectively in 23 CD patients. Results were compared with data of CD patients reported in three prior large series. Kaplan Meier survival analysis including log rank test was performed for pooled data of 82 CD patients (study cohort and literature patients).

**Results:**

Onset of symptoms was between 0 and 6 months. Psychomotor development of patients was limited to abilities that are usually gained within the first year of life. Macrocephaly became apparent between 4 and 18 months of age. Seizure frequency was highest towards the end of the first decade. Ethnic background was more diverse than in studies previously reported. A CD severity score with assessment of 11 symptoms and abilities was developed.

**Conclusions:**

Early hallmarks of CD are severe psychomotor disability and macrocephaly that develop within the first 18 months of life. While rare in the first year of life, seizures increase in frequency over time in most patients. CD occurs more frequently outside Ashkenazi Jewish communities than previously reported. Concordance of phenotypes between siblings but not patients with identical *ASPA* mutations suggest the influence of yet unknown modifiers. A CD severity score may allow for assessment of CD disease severity both retrospectively and prospectively.

**Supplementary Information:**

The online version contains supplementary material available at 10.1186/s13023-020-01659-3.

## Background

Canavan disease (CD) is a genetic degenerative brain disorder caused by deficiency of the enzyme aspartoacylase (ASPA). The loss of ASPA activity results in an accumulation of N-acetylaspartic acid (NAA) in the brain and other parts of the body [1]. NAA is suspected to function as a molecular water pump [2], leading to fatal brain disease for which there is currently no effective treatment. CD is diagnosed by detection of elevated NAA in urine or blood or in brain by proton MR spectroscopy [3], as well as by *ASPA* mutation analysis [4]. Histologically the disease is characterized by insufficient myelination and progressive spongy degeneration of the brain white matter [5, 6]. The perinatal period is often uneventful and subtle changes during early infancy may escape attention. The evolution of symptoms distinguishes Canavan disease from static encephalopathies, such as cerebral palsy. To enable early diagnosis first symptoms and clinical signs specific to CD need to be recognized.

While CD is presently incurable, experimental therapies are in development [7]. Their implementation requires an understanding of the optimal window of intervention. Detailed knowledge of early symptoms and the natural course of the disease provide a framework for trial design. Here, we identify early features of CD and propose a CD severity score based on a comprehensive study of the clinical course of 23 CD patients, including retrospective longitudinal data, in comparison with data of CD patients reported in the literature.

## Results

### Patient population

Most common country of residence was Germany, followed by the U.S. Twenty of 23 patients reported a non-Jewish ethnic background. In most patients birth and perinatal development was uneventful, Additional file 1: Table 1. Genetic information of 16 patients was available, Table 1. Siblings of two families presented a similar course of disease.

**Table 1:** Genetic information of 16 CD patients (Study group)

Patient	cDNA variant	Protein variant	Type of mutation	ACMG classification of variant (pathogenic, likely pathogenic, VOUS)	Previously reported (yes/no)
1/1	c.[914C > A];[914C > A]	p.[Ala305Glu];[Ala305Glu]	Missense/missense	Likely pathogenic/likely pathogenic	Yes/yes
2/1	c.[854A > C];[854A > C]	p.[Glu285Ala];[Glu285Ala]	Missense/missense	Likely pathogenic/likely pathogenic	Yes/yes
3/1	c.[914C > A];[914C > A]	p.[Ala305Glu];[Ala305Glu]	Missense/missense	Likely pathogenic/likely pathogenic	Yes/yes
4/1	c.[859G > A];[914C > A]	p.[Ala287Thr];[Ala305Glu]	Missense/missense	Pathogenic/likely pathogenic	Yes/yes
5/1	c.[854A > C];[914C > A]	p.[Glu285Ala];[Ala305Glu]	Missense/missense	Likely pathogenic/likely pathogenic	Yes/yes
6/1	c.[541C > A];[426C > A]	p.[Pro181Thr];[Tyr142*]	Missense/nonsense	Likely pathogenic/pathogenic	Yes/no
7/1^a^	c.[634 + 1G > T];[634 + 1G > T]	p.[?];[?]	Splice site/splice site	Pathogenic/pathogenic	Yes/yes
7/2 ^a^	c.[634 + 1G > T];[634 + 1G > T]	p.[?];[?]	splice site/splice site	pathogenic/pathogenic	Yes/yes
8/1	c.[914C > A];[362A > T]	p.[Ala305Glu];[Asn121Ile]	Missense/missense	Likely pathogenic/likely pathogenic	Yes/yes
9/1	c.[878_880delAAG];[?]	p.[Glu293del];[?]	In frame del/?	Likely pathogenic/?	Yes/?
10/1	c.[885dupT];[?]	p.[Ala296Cysfs*5];[?]	Frameshift/?	Pathogenic/?	No/?
11/1	c.[838C > T];[79G > T]	p.[Pro280Ser];[Gly27*]	Missense/nonsense	Pathogenic/pathogenic	Yes/yes
12/1	c.[296delT];[296delT]	p.[Leu99Tyrfs*38];[Leu99Tyrfs*38]	Monsense/nonsense	Pathogenic/pathogenic	No/no
13/1	c.[854A > C];[693C > A]	p.[Glu285Ala];[Tyr231*]	Missense/missense	Likely pathogenic/pathogenic	Yes/yes
14/1 ^a^	c.[914C > A];[914C > A]	p.[Ala305Glu];[Ala305Glu]	Missense/missense	Likely pathogenic/likely pathogenic	Yes/yes
14/2 ^a^	c.[914C > A];[c.914C > A]	p.[Ala305Glu];[Ala305Glu]	Missense/missense	Likely pathogenic/likely pathogenic	Yes/yes

^a^Siblings

### Onset of CD disease and diagnosis

**Fig. 1:**
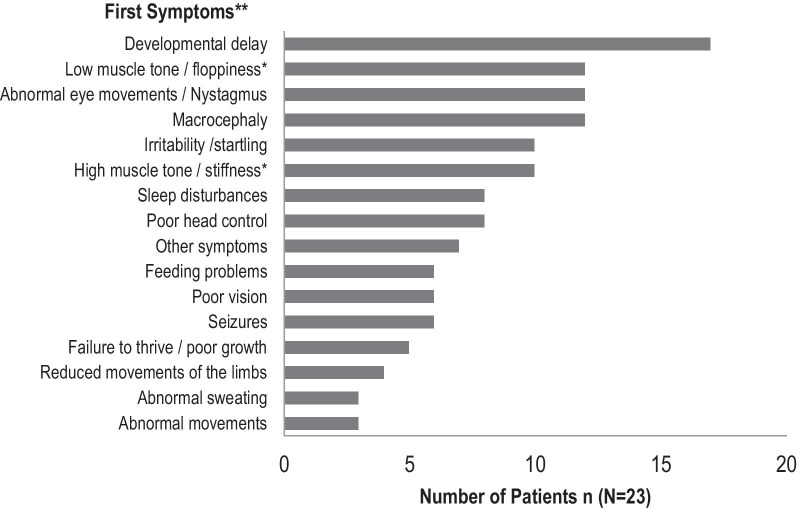
Clinical features at onset of disease in the cohort of 23 CD patients. Bars indicate the number of patients that developed the symptom. ** 23/23 patients in the study group showed a combination of 6 symptoms in median (range 2–12 symptoms) at onset of disease. * 4/23 patients showed a combination of low muscle tone (axial hypotonia) and high muscle tone (appendicular hypertonia). 6/23 patients showed only high muscle tone. 8/23 patients showed only low muscle tone

All patients showed 2–12 symptoms within the first 6 months of life such as developmental delay (17/23), macrocephaly (12/23) and abnormal eye movements (12/23), Fig. 1. Diagnosis was made between 0 and 29 months.

### Developmental milestones and developmental regression

**Fig. 2:**
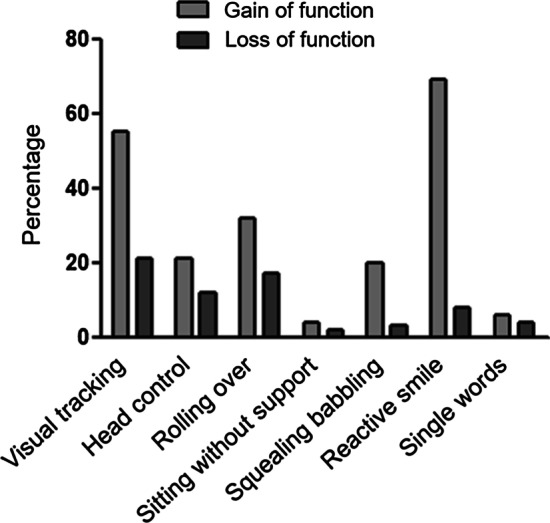
Cumulative percentage of gain and loss of functions (study cohort and literature patients)

All patients were reported to be able to hear. More than fifty percent of the patients gained the ability to visually track, only 9/23 were able to roll over (Fig. 2). Only 3/23 patients of our study cohort acquired further fine motor skills such as the ability to draw or scribble. Fine motor skills were preserved for a longer median time span than gross motor abilities (Table 2). Thirteen percent of our patients learned to speak single words, none could speak in sentences. None of the patients in our cohort was toilet trained (Table 2).

**Table 2:** Gain and loss of developmental milestones in CD patients: study cohort versus literature

Milestones of psychomotor development	Patients who gained function, *n (N*) percentage	Patients who lost function, *n (N*) percentage	Age of gain, median (range), mo	Age of loss, median (range), mo	Normal age of gain (DDST II), ranges, mo	Patients who gained function, Traeger et al., n (N) percentage	Patients who lost funktion, Traeger et al., n (N) percentage	Age of gain, Traeger et al., median (range), mo	Age of loss, Traeger et al., median (range), mo
*Hearing/Vision*									
Visual tracking	12/23 52%	7/23 30%	4 (0–15)	14 (1–132)	0–4	33/59 56%	10/59 17%	4 (1–18)	12 (4–44)
Hearing	23/23 100%	0/23 0%	0 (0–0)		0				
*Gross Motor Skills*									
Head control	6/23 26%	2/22 9%	6 (2–10)	6 (6–6)	1.5–4	11/59 19%	8/59 14%	6 (1–24)	11.5 (2–180)
Rolling over	9/23 39%	7/22 32%	6 (2–12)	11 (4–120)	4.25–5.5	17/59 29%	7/59 12%	6 (2–24)	11.5 (6–72)
Sitting with support	6/23 26%	2/23 9%	5.5 (4–8)	15 (6–24)					
Sitting without support	2/23 9%	1/23 4%	11.5 (11–12)	120	6.75–8	1/59 2%	1/59 2%	10	60
Crawling	0/22	–	–	–		1/59 2%	1/59 2%	10	72
Standing with support	1/23 4%	1/22 5%	13	132	6.75–10.5				
Standing without support	0/23	–	–	–	11.25–16				
Walking with support	3/23 13%	2/23 9%	18 (15–19)	84 (36–132)					
Walking	0/23	–	–	–	12.25–16				
*Fine Motor Skills*									
Reaching for an object	7/22 30%	2/22 9%	7 (4–10)	69 (6–132)	3.5–6				
Voluntary hand function	7/23 30%	1/22 5%	7 (1–18)	6					
Transfer an item	5/23 22%	4/23 17%	9 (4–18)	31 (6–132)	5–8.75				
Scribbling/drawing	3/23 13%	2/23 9%	9	78 (24–132)	12.25–21				
*Language/Other*									
Imitating noises	3/22 13%	2/22 9%	11 (10–12)	96 (60–132)	6.5–12.75				
Squealing /babbling	5/16 31%	2/15 13%	8 (4–55)	156 (132–180)	1.5–3.75	10/59 17%	0/59	5 (1–18)	–
Understand language	11/23 48%	1/22 5%	11 (7–24)	7	–				
Reactive smiling	7/11 64%	2/11 18%	5 (1–14)	205.5 (132–281)	0–1.5	43/60 72%	4/60 7%	3 (0–36)	13.5 (4–150)
Communicate	8/22 36%	1/22 5%	11 (4–24)	180*	–				
Single words	3/23 13%	2/23 9%	24 (12–24)	93.5 (48–139)	10–14.5	2/59 3%	1/59 2%	23.5 (11–36)	180
Single sentences	0/23	–	–						
Count to five	1/23 4%	1/23 4%	60*	132*					
Independent eating	2/23 9%	0/23	11	–					
Toilet trained	0/23	–	–	–

**Table 3:** Canavan disease rating score

Symptoms/neurological domain	Deficit absent/Within normal limits/(score = 0)	Deficit present intermittently or mild (score = 1)	Deficit present constantly or pronounced (score = 2)
Epileptic Seizures^a^			
Floppiness (truncal hypotonia)			
Spasticity (appendicular hypertonia)			
Macrocephaly^b^			
Feeding^c^			
Language^d^			
Responsive social smile			
Visual tracking			
Head control			
Reaches for objects			
Sits without support			

Score 0 if findings are normal/appropriate to age.

^a^Epileptic seizures: Score 0 if no epileptic seizures, not taking antiepileptic drug/s (AED). Score 1 if 1–2 unprovoked seizures/year, not taking AED/s OR no seizures but on 1 AED. Score 2 if > /= 3 > /= 3 unprovoked seizures /year OR no seizures but requires more than 1 AED

^b^Macrocephaly: Score 1 if head growth is accelerated (absolute head size is still within normal range but > 2 or more major percentiles (5, 10, 25, 50, 75, 90, 95 P) are crossed). Score 2 if head size is above 97th Percentile

^c^Feeding: Score 1 if oral feeding is possible but aspiration occurs and/or weight gain is insufficient (patient losing weight or crossing insufficient (patient losing weight or crossing of 2 or more major percentiles (5, 10, 25, 50, 75, 90, 95 P) of his bodyweight). Score 2 if tube feeding is necessary

^d^Language delay: Score 1 if some vocalization besides crying. Score 2 if no vocalization besides crying

Minimum: 0 Points (appropriate for age)

Maximum: 22 points (severely affected)

### Neurological symptoms

Spasticity was most frequent. Hyperexcitability to noise was reported for 13/22 patients and lost over time by 8 patients. At onset of disease, abnormal eye movements such as nystagmus or inattentive gaze were reported in 12/23 patients, at follow up in 21/23 patients.

Despite early onset of symptoms, 2/3 of CD patient did not have seizures in the first year of life. Longitudinal data showed that seizure frequency increased significantly over time and tended to be higher towards the end of the first decade of life, Additional file 2: Fig. 1. The majority of seizures (10/14) required anticonvulsant medication.

Longitudinal data of head circumference were provided by 19/23 patients. Macrocephaly occurred between 0 and 19 months with a slightly earlier median age at onset in girls (7 months) than in boys (8.5 months), Additional file 2: Fig. 2.

### Other health problems

Neurological symptoms, gastrointestinal and pulmonary problems were frequently reported. Only 2/23 patients gained the ability to eat independently. A gastric tube was placed in 10/22 patients.

### Off label use of medications

Eleven of 23 patients reported temporary usage of non-approved medication such as lithium, acetazolamide, calcium acetate, amoxicillin, sodium succinate, acetyl-L-carnitine etc. (Additional file 1: Table 3).

Few patients (3/23) reported improved alertness and mood stabilizing effects with lithium. None of the patients had improvement in psychomotor abilities, neurological symptoms or head growth. Reported side effects included gastrointestinal symptoms or decreased alertness. A few of the patients’ caregivers (3/11) attributed positive effects to the experimental treatment. Negative side effects, which mainly concerned the gastrointestinal system, were reported for 5/11 patients who received medication that was meant to ameliorate the course of CD. Half of the patients (5/10) who received anticonvulsants, required multiple drugs to achieve symptom control.

### Data analysis of patients from the literature

Data analysis of patients from the literature showed a slightly later median onset of first symptoms (3 months, range 0–18). Data on developmental milestones showed no difference between patients reported in the literature and our study cohort (Table 2). In the literature 57% of patients developed seizures [10, 11] a similar percentage as in our study cohort. Onset of seizures was only slightly later, Additional file 1: Table 2. 91% of CD patients reported in the literature showed macrocephaly (median age at onset was 5 months, range 0–60) [10] compared to 100% of our study cohort. Gastric tubes were placed for 52% of the patients in the literature cohort [10], with median age of onset at 39 (range 6–228) months, which is significantly later than in patients in our study group (median age of onset 22, range 10–179 months).

### Survival analysis with comparison of patient groups (pooled data)

**Fig. 3:**
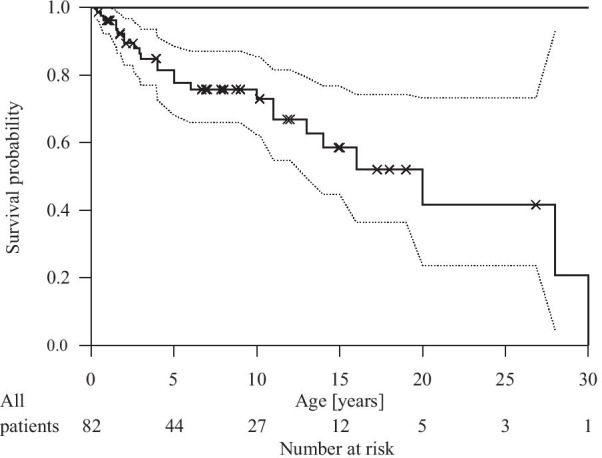
Kaplan Meier survival probability with confidence interval (dotted line) for 82 CD patients, pooled data: study cohort (N = 23) & literature cohort (N = 59) /Traeger et al.

Five of 23 patients in our study cohort and 21/59 patients in the literature group [10] died, a total of 26/59. 73% of the CD patients reached the age of 10 years (Fig. 3). There was no significant difference between survival rates of patients reported in the literature and patients in our study cohort. Neither seizures nor basic psychomotor skills (visual tracking & head control) within the first two years of life had a statistically significant influence upon survival.

### Canavan disease severity score

To assess disease burden in CD we chose 11 pertinent symptoms that affect CD patients. Based on the findings of our natural history study we selected items that would best represent severity of disease in CD. Items were reviewed and modified for content validity, clarity, and face validity. They included common symptoms as macrocephaly, spasticity and seizures and also encompassed domains of common milestones gained and lost. The scoring was based on points that were given depending on the absence (score = 0) or intermittent (score = 1) or constant presence (score = 2) of a symptom or abnormality. This resulted in a sum of 0–22 points. This score is intended to be used prospectively and retrospectively (analyzing medical records and CD questionnaires and findings on medical examination) Table 3. A pilot study assessing interrater variability revealed that the level of agreement was 96% (74 of 77 scoring points).

## Discussion

This study describes the clinical course of 23 CD patients in detail and compares it with data of 90 patients reported in the literature. We further report a heterogeneity of ethnic background in CD patients that has not been published to date.

In our cohort, CD patients showed first symptoms at 2–4 months of age. At first onset most families reported a combination of up to twelve different symptoms, Fig. 1.

Only 3/23 patients acquired skills consistent with normal development in healthy children. These less severely affected patients learned to sit without support, developed basic fine motor skills (reaching for an object, transferring an item and scribbling) and acquired the ability to speak single words. Our data as well as data from the literature showed, that if patients did not acquire sitting by the age of 12 months, it was unlikely that they would ever gain it.

All patients, even those who gained a higher number of more advanced abilities, lost these skills during their course of disease. Our data show that fine motor skills were retained for a longer time than gross motor skills. Language comprehension was more commonly present in CD patients than expressive language and was retained for a longer time span. None of our patients was ever able to speak in full sentences.

While not all CD patients suffered from epilepsy during the first years of life, over the first decade seizure prevalence increased, with all CD patients suffering from seizures beyond 10 years of age. Management of neurological symptoms such as seizures and spasticity are often complicated by the need for multiple antispasmodic or antiepileptic drugs. There was no evidence that usage of experimental drugs showed any distinct benefit. Only lithium was reported to improve alertness and to contribute to mood stabilization. It had no effect upon the disease course or the number of abilities gained.

CD manifests in early childhood and leads to severe disability and shortened life expectancy. Recent reports state that NAA itself is not required for myelination [13]. Rather, high levels of NAA, as found in CD, are thought to cause a toxic imbalance of brain water homeostasis [14] and oxidative stress [15, 16] which contributes to characteristic formation of vacuoles within the myelin, causing severe neurological symptoms. After an unremarkable perinatal course, affected children typically stagnate in their psychomotor milestones within the first six months of life. Only few CD patients progress beyond the developmental age of approximately 3 months.

Our survival analysis showed that about 73% of CD patients reach the age of ten years which extends survival beyond what had previously been reported [17]. Lyon et al. had listed survival limited to three years with only rare exceptions to 10 years of life. We think prolonged survival may be due to improved care standards over time.

Our analysis of longitudinal data revealed details on the onset of macrocephaly during the first year of life (mean age for girls: 7 and for boys: 8.5 months) with a plateau occurring around the age of 18 months. Onset and frequency of macrocephaly documented for our study group was comparable to data from the literature. A larger cohort of CD patients is needed to determine whether gender impacts the age at onset of macrocephaly.

Macrocephaly in combination with early developmental stagnation is the hallmark of CD and allows clinical differentiation from many other infantile encephalopathies, such as cerebral palsy or other neurodegenerative disorders such as Krabbe disease. Within the group of neurodegenerative diseases with macrocephaly, developmental delay, affected brain white matter and visual problems, CD needs to be distinguished from genetic encephalopathies such as GM1 or GM2 gangliosidosis, PTEN (Phosphatase and tensin homolog protein diseases), megalencephalic leukoencephalopathy with subcortical cysts, Alexander disease or glutaric aciduria. Diagnosis can then easily be accomplished by measuring NAA levels in urine or on brain MRS.

To define hallmarks of the disease for endpoints of future clinical trials one first must consider that most CD patients never attain motor and language milestones. Next, potential endpoints should be chosen that can be compared across a large number of CD patients, such as the ability of visual tracking that 12/23 patients gained, and 7 CD patients subsequently lost again. Considering that only two patients were reported to suffer from optic nerve atrophy, we conclude that visual impairment is more commonly caused by white matter disease affecting the visual pathways.

Patients with CD suffer from several concomitant disabilities. The result that gastric tubes of literature patients [10] being implanted significantly later (39 months versus 22 months within our study cohort) is likely related to improved standards of palliative care over time. We could not find any evidence that gaining early milestones in psychomotor development such as head control and visual tracking, was associated with prolonged survival.

All CD patients were reported by caregivers and medical records to be able to hear at birth and throughout the course of disease. This stands in contrast to findings of impaired hearing reported in the Canavan mouse model [14]. Interestingly, acoustic startle was noted by more than half of the caregivers. This contradicts Lyon et al. [17] who mentioned that CD patients show tonic spasms by acoustic stimuli or handling rather than a repetitive acoustic startle as seen in Tay-Sachs disease. Our clinical impression is that both tonic spasms as well as a startle response can be seen in children with CD.

Perhaps due to the small numbers of and the fact that patients with a milder phenotype are not coming to our attention, we could not demonstrate a genotype phenotype correlation within our study cohort. However, for less severely affected patients of our cohort we observed molecular genetic findings such as p.Glu285Ala;p.Ala305Glu/p.Ala305Glu;p.Ala305Glu/p.Ala287Thr;p.Ala305Glu. These patients achieved and sustained a higher number of abilities and more complex functions. We also observed similar courses of the disease among siblings. Individual reports of ambulatory patients without macrocephaly suggest that milder phenotypes may be more abundant [18].

Limitations of our study were that cognitive testing by standard instruments was not possible due to the severity of disease in our study population. Additionally, there were only very limited MRI data due to the need for sedation that was not justified in a natural history without intervention.

To distinguish milder from more severe courses of the disease, we chose 11 pertinent symptoms that serve as a benchmark for disease progression and may be critical to future clinical trial design. Disease severity shall be ascertained retrospectively and prospectively. First assessments of the CDRS found an interrater variability of 96%. Further natural history studies are needed to validate the CDRS and optimize its performance in prospective studies.

## Conclusions

Canavan Disease remains one of the most devastating inherited neurological disorders. Macrocephaly and developmental delay within the first year of life and subsequent loss of skills mark the clinical course with seizures increasing towards the end of the first decade. Our results suggest a window of intervention within the first two years of life. Arrested development of many CD patients after 12 months of age imply that intervention within the first 12 months of life may have the largest impact.

## Methods

Patients were recruited at our outpatient clinics (Department of Pediatrics, University Medical Center Hamburg Eppendorf, Hamburg, Germany, and the Department of Neurology, MGH, Harvard Medical School, Boston, USA, supported by cooperating partners and patient organizations as NTSAD, Myelin Project Germany and ELA Germany) over a 6-year period by sending out questionnaires to 30 caregivers of CD patients. Data on the natural course of 23 CD patients (11male,12female) were collected retrospectively. Data sources consisted of the patients’ medical records and standardized patients’ questionnaires. All data were anonymized, entered and processed within the web-based Hamburg Leukodystrophy Database. All entered data were cross-checked by two experienced data managers.

Only those patients were included, whose diagnosis was confirmed by urine analysis of NAA and/or by *ASPA* gene mutation analysis. Onset of CD was defined as time when first symptoms were observed by the parents and/or documented in the patients’ medical history records.

To describe acquisition and regression of psychomotor developmental milestones, we analyzed the median age of milestone acquisition, as well as median age of regression of skills for each psychomotor ability listed in the CD questionnaire. The categories were gross and fine motor skills, vision, hearing and language and other abilities listed in Table 1 and were based on clinical experience with CD and other early infantile neurodegenerative diseases such as Krabbe leukodystrophy. Whenever reasonable and available, psychomotor abilities were compared with control data from the Denver Developmental Screening Test II/DDST II [8]. If available, psychomotor skills and neurological symptoms were compared with the published literature of CD patients.

In order to describe neurological symptoms that are typically associated with CD, we asked for age of onset of spasticity, abnormal eye movements, seizures, dystonic hyperkinetic movements and irregularities of the eye ground. Caregivers were asked to document medications necessary to control the above-mentioned symptoms.

To analyze CD patients’ seizure history, we collected longitudinal data and asked for annual seizure frequency. Types of seizures were not specified. Seizure frequency was classified into 4 categories: no seizure/year, 1–2 seizures/year, less than 12 seizures/year and more than 12 seizures/year based on experience with other infantile neurodegenerative diseases.

To evaluate onset and course of macrocephaly, longitudinal data of head circumference in CD patients was collected and compared with reference percentiles, Robert Koch Institute, KiGGS [9]. Macrocephaly was defined as a head circumference larger than 97th percentile.

### Review of literature/literature CD patients

We searched Pubmed literature from 1976 to 2016 with key words “Canavan” (192 results) and “Van Bogaert-Bertrand” (21 results) for articles reporting the clinical course of CD patients. Data of three articles [10–12] reporting on larger patient cohorts (N > 10) were included. Frequency and median age of milestone acquisition and regression of single psychomotor skills, onset of disease and neurologic symptoms, as well as follow up/survival time was calculated for the literature group and compared with the data from our study group.

For analysis of disease onset, data of all three articles were used, summarizing up to 90 patients in the literature. For analysis of seizure onset, data of 74 patients reported by Ozand et al. [11] and Traeger et al. [10] were analysed and used for comparison with our study data. For analysis of psychomotor development, macrocephaly and gastric tube placement, data of 60 patients reported by Traeger et al. [10] were included.

### Statistical methods/survival analysis

We pooled data of our study cohort and a cohort of 60 CD patients reported by Traeger et al., summarizing a total number of 82 patients. We performed Kaplan Meier survival analysis to investigate differences in survival rates between the study cohort and literature group. A Log Rank Test was performed to test if there were significant differences between patient groups.

To investigate the influence of certain neurological symptoms, clinical complications or development of psychomotor skills upon survival, we selected those patients from both groups (literature and study group) who were observed for longer than one year. We analyzed whether development of seizures, the need for gastric tube placement or development of basic psychomotor skills (visual tracking & head control) within the first two years of life had an influence on survival of CD patients.

A pilot study to assess interrater variability of the CDRS was performed. Two independent physicians (one knowing the subjects, the other one not familiar with the patients) reviewed medical letters from different time points.

## Supplementary information

**Additional file 1:** **Table 1**. List of Patients. **Table 2** Neurologic symptoms: Frequency and onset of neurologic symptoms of CD patients (study group) sorted by age at onset of symptoms (ascending). * Traeger et al. [1]; Nystagmus: n/N = 51/59, median of age of onset 3 months (range 0–60 months), **Traeger et al. [1], Ozand et al. [2]: Seizures: n/N = 42/74, median of age of onset 19 months (range 0–180 months). **Table 3**. Medication received by study patients: Positive effects of the experimental treatment were attributed by 3/11 of the patients’ caregivers. Negative side effects, which mainly concerned the gastrointestinal system, were reported for 5/11 patients who received medication that was meant to ameliorate the course of CD. 50% (5/10) of the patients that received anticonvulsive drugs, needed combined use of multiple drugs in order to control the symptoms.

**Additional file 2.** **Figure 1**: Seizure frequency of Canavan patients over the course of disease. Seizure frequency of CD patients over the decade of life is shown. Seizure Frequency is classified in 4 groups: no seizures/year, 1-2 seizures/year, <12 seizures/year, >12 seizures/year. Percentage means: Number of patients with a certain seizure frequency / number of all study participant of this age. **Figure 2**: Head circumference of 9 female and 10 male patients with Canavan disease within the first 6.5 years of life. Bold lines stand for patient’s data series. Dotted lines indicate males, continuous lines females. Local regression (loess) and 95% confidence interval of loess was estimated. Thin lines and grey area indicates reference percentiles for head circumference of girls and boys (Robert-Koch-Institute, KIGGS).

## Data Availability

The datasets used and/or analyzed during the current study are available from the corresponding author on reasonable request.

## Abbreviations

ASPA: Aspartoacylase
CD: Canavan disease
DDST II: Denver developmental screening test II
NAA: N-acetyl aspartate
