# Supplementary material for: The natural history of Canavan disease: 23 new cases and comparison with patients from literature
Source: Orphanet J Rare Dis. 2021 May 19;16:227. doi: 10.1186/s13023-020-01659-3 (PMC8132415; doi:10.1186/s13023-020-01659-3)
Supplement: Supplementary file 1 — Additional file 1: Table 1. List of Patients. Table 2 Neurologic symptoms: Frequency and onset of neurologic symptoms of CD patients (study group) sorted by age at onset of symptoms (ascending). * Traeger et al. [1]; Nystagmus: n/N = 51/59, median of age of onset 3 months (range 0–60 months), **Traeger et al. [1], Ozand et al. [2]: Seizures: n/N = 42/74, median of age of onset 19 months (range 0–180 months). Table 3. Medication received by study patients: Positive effects of the experimental treatment were attributed by 3/11 of the patients’ caregivers. Negative side effects, which mainly concerned the gastrointestinal system, were reported for 5/11 patients who received medication that was meant to ameliorate the course of CD. 50% (5/10) of the patients that received anticonvulsive drugs, needed combined use of multiple drugs in order to control the symptoms. [file 13023_2020_1659_MOESM1_ESM.docx]

**Supplement Table 1 List of Patients**

|  | Percentage of Study patients, (N=23) | Percentageof literature patients (1) n / N |
| --- | --- | --- |
| **Jewish Ancestry** |  |  |
| No Jewish Ancestry | 87% (20 / 23) | 51% (46 / 90) |
| Jewish Ancestry | 13% (3 / 23) | 49% (44 / 90) |
|  |  |  |
| **Patients’ Country of living** |  |  |
| Germany | 57% (13 / 23) |  |
| USA | 26% (6 / 23) |  |
| Spain | 4% (1 / 23) |  |
| Russia | 4% (1 / 23) |  |
| Belarus | 4% (1 / 23) |  |
| Australia | 4% (1 / 23) |  |
|  |  |  |
| **Survival** |  |  |
| Patient deceased | 22% (5 / 23) | 36% 21 / 59 |
| Median age at death, months | 24 (4 - 35) |  |
|  |  |  |
| **Perinatal information** | Median (range) N |  |
| All patients | 23 |  |
| Delivery at full term | 21 |  |
| Weight, g | 3493 (2190 - 4356) |  |
| Length, cm | 52 (44 -59) |  |
| Head circumference, cm | 35 (32 - 37) |  |
| Apgar 5 min | 10 (9 - 10) |  |
| Apgar 10 min | 10 (7 - 10) |  |

**Supplement Table 2 Neurologic symptoms**

|  | Study group Percentage n/(N) | Age of onset median (range), mo |
| --- | --- | --- |
| Spasticity | 95%, 19 / 20, | 4 (0 - 28) |
| Dystonic hyperkinetic movements | 27%, 6 / 22 | 4 (4 - 84) |
| Abnormal eye movements | 91%, 21 / 23 | 4.5 (0 - 127) |
| Irregularities eye ground | 14%, 3 / 22 | 6 (4 - 6) |
| Seizures** | 61%, 14 / 23 | 9.5 (1 - 60) |

Frequency and onset of neurologic symptoms of CD patients (study group) sorted by age at onset of symptoms (ascending). * *Traeger et al [1]*; Nystagmus: n/N = 51 / 59, median of age of onset 3 months (range 0 – 60 months), ** *Traeger et al[1], Ozand et al [2]*: Seizures: n/N = 42 / 74, median of age of onset 19 months (range 0 – 180 months).

**Supplement Table 3 Medication received by study patients**

| Antispastic medication | Nr. of pat.:12 | Anticonvulsive medication | Nr of pat: 12 | Additional symptomatic medication | Nr. of pat:  12 | Experimental medication | Nr. of pat.  11 |
| --- | --- | --- | --- | --- | --- | --- | --- |
| Baclofen | 11 | Phenobarbital | 4 | Reflux control | 6 | Calcium-acetate | 7 |
| Botox | 5 | Levetiracetam | 4 | Asthma control/ airway dilatation | 5 | Lithium | 5 |
| Clona-zepam | 2 | Valproate | 2 | Laxatives | 5 | Acetazolamide | 5 |
| other | 5 | Lamotrigine | 2 | Tranquilizer | 3 | Amoxicillin | 4 |
|  |  | other | 4 | Antibiotics | 3 | Sodium Succinate | 3 |
|  |  |  |  | Anticholinergics | 2 | Acetyl-L-Carnitin | 1 |
|  |  |  |  |  |  | Sodium Bi-carbonate | 1 |
| use of > 1 drug | 3 | use of >1 drug | 5 |  |  |  |  |

Positive effects of the experimental treatment were attributed by 3 / 11 of the patients’ caregivers. Negative side effects, which mainly concerned the gastrointestinal system, were reported for 5 / 11 patients who received medication that was meant to ameliorate the course of CD. 50% (5 / 10) of the patients that received anticonvulsive drugs, needed combined use of multiple drugs in order to control the symptoms.
