# Supplementary figures and images for: The natural history of Canavan disease: 23 new cases and comparison with patients from literature
Source: Orphanet J Rare Dis. 2021 May 19;16:227. doi: 10.1186/s13023-020-01659-3 (PMC8132415; doi:10.1186/s13023-020-01659-3)

**Supplement Figure 1**

**
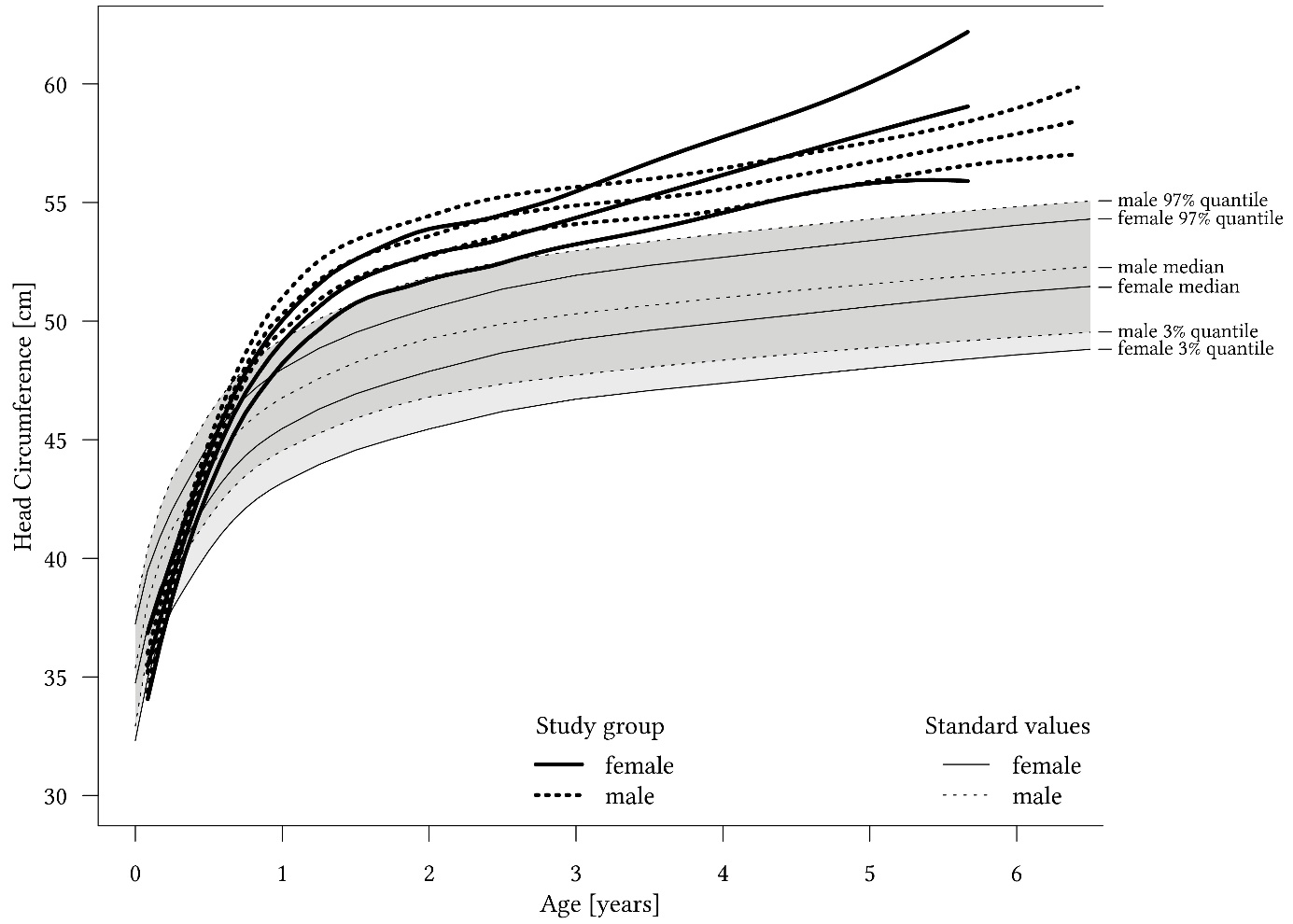
**

**Supplement Figure 2**

Supplement: Supplementary file 2 — Additional file 2. Figure 1: Seizure frequency of Canavan patients over the course of disease. Seizure frequency of CD patients over the decade of life is shown. Seizure Frequency is classified in 4 groups: no seizures/year, 1-2 seizures/year, <12 seizures/year, >12 seizures/year. Percentage means: Number of patients with a certain seizure frequency / number of all study participant of this age. Figure 2: Head circumference of 9 female and 10 male patients with Canavan disease within the first 6.5 years of life. Bold lines stand for patient’s data series. Dotted lines indicate males, continuous lines females. Local regression (loess) and 95% confidence interval of loess was estimated. Thin lines and grey area indicates reference percentiles for head circumference of girls and boys (Robert-Koch-Institute, KIGGS). [file 13023_2020_1659_MOESM2_ESM.docx]
